# Accidental Impurities in Epitaxial Pb(Zr_0.2_Ti_0.8_)O_3_ Thin Films Grown by Pulsed Laser Deposition and Their Impact on the Macroscopic Electric Properties

**DOI:** 10.3390/nano11051177

**Published:** 2021-04-29

**Authors:** Georgia Andra Boni, Cristina Florentina Chirila, Viorica Stancu, Luminita Amarande, Iuliana Pasuk, Lucian Trupina, Cosmin Marian Istrate, Cristian Radu, Andrei Tomulescu, Stefan Neatu, Ioana Pintilie, Lucian Pintilie

**Affiliations:** National Institute of Materials Physics, Atomistilor 405A, 077125 Magurele, Romania; andra.boni@infim.ro (G.A.B.); dragoi@infim.ro (C.F.C.); stancu@infim.ro (V.S.); amarande@infim.ro (L.A.); iuliana.pasuk@infim.ro (I.P.); Lucian.Trupina@infim.ro (L.T.); cosmin.istrate@infim.ro (C.M.I.); cristian.radu@infim.ro (C.R.); andrei.tomulescu@infim.ro (A.T.); stefan.neatu@infim.ro (S.N.); ioana@infim.ro (I.P.)

**Keywords:** thin films, ferroelectric, impurities, electric properties, pulsed laser deposition

## Abstract

Structural and electrical properties of epitaxial Pb(Zr_0.2_Ti_0.8_)O_3_ films grown by pulsed laser deposition from targets with different purities are investigated in this study. One target was produced in-house by using high purity precursor oxides (at least 99.99%), and the other target was a commercial product (99.9% purity). It was found that the out-of-plane lattice constant is about 0.15% larger and the *a* domains amount is lower for the film grown from the commercial target. The polarization value is slightly lower, the dielectric constant is larger, and the height of the potential barrier at the electrode interfaces is larger for the film deposited from the pure target. The differences are attributed to the accidental impurities, with a larger amount in the commercial target as revealed by composition analysis using inductive coupling plasma-mass spectrometry. The heterovalent impurities can act as donors or acceptors, modifying the electronic characteristics. Thus, mastering impurities is a prerequisite for obtaining reliable and reproducible properties and advancing towards all ferroelectric devices.

## 1. Introduction

Ferroelectric materials with perovskite structures, as for example Pb(Zr,Ti)O_3_ (PZT) or (Ba,Sr)TiO_3_ (BST) solid solutions, have been intensively studied due to their impressive potential for applications in various domains ranging from domestic devices like burglar alarms or ultrasonic cleaners up to high-tech applications like non-volatile memories, infrared imaging, photovoltaics and energy harvesting, or agile antennae for microwave telecommunications. All these applications rely on the unique property of ferroelectrics to possess a spontaneous polarization that can be controlled with an applied external electric field, giving a hysteresis loop that is at the base of memory applications, or can be varied with temperature or mechanical stress, leading to pyroelectric and piezoelectric effect [1,2,3,4,5,6,7,8,9]. PZT or BST materials can be prepared as bulk ceramics or in form of thin films. In the latter case, a variety of techniques can be used to obtain the films, from simple and low cost chemical bath deposition methods (e.g., sol–gel), to more sophisticated methods requiring special deposition chambers with vacuum equipment, as is the case for radio-frequency (RF) magnetron sputtering or pulsed laser deposition (PLD) [10,11,12]. In all cases, the ferroelectric materials are obtained starting from certain precursor chemicals. In the case of the bulk ceramics, the precursors are, usually, oxides of the component cations [13], while in the case of the thin films, the precursors can be organic compounds containing the cations, as in the case of sol–gel, or ceramic targets, as in the case of RF sputtering of PLD. All these precursors have a certain purity and may contain a small amount of impurities that may affect or not the macroscopic electric properties of ferroelectrics like PZT or BST.

PLD is a method that allows for the growth of epitaxial ferroelectric films; thus it is increasingly used for the study of these materials because it allows elimination of many extrinsic contributions to the macroscopic electric properties (e.g., grain boundaries) and allows integration of epitaxial ferroelectric layers with semiconductor technology by a proper selection of the buffer layer, as for example epitaxial SrTiO3 (STO) on Silicon (Si) [14,15,16]. As mentioned above, ceramic targets are used for PLD growth, targets that can be purchased from the market (commercial targets) or can be manufactured in-house by using the standard ceramic technology (milling the powders of precursor oxides, following by calcination annealing to initiate the solid state reaction, milling again, cold pressing, and final sintering annealing to obtain the dense ceramic target) [17]. Whether commercial or made in-house, the properties of the films can be affected by the amount of impurities from the target, especially if these impurities act as donors or acceptors, influencing the density of free carriers and impacting the magnitude of the leakage current, polarization switching, and other macroscopic properties relevant for various applications. The role of dopants in ferroelectrics has been widely studied, especially in ceramics, but also in thin films [17,18,19,20,21,22]. However, it appears that no special attention was given to compare the results with a reference sample, assumed to be as pure as possible, following a similar path as for semiconductors (e.g., in the case of Si, first the effort was made to obtain a pure material and then to dope it in a controlled way to obtain clear n or p type conduction [23,24]). Therefore, if one intends to obtain n or p type doping in ferroelectric thin films, then one has to obtain first a pure ferroelectric sample, considering that any unwanted impurity from the precursor materials or from a target can have a possible detrimental impact on the macroscopic electrical properties.

Following the literature, although it seems to be a trivial problem, it appears that the relation between target purity and the electric properties of epitaxial films grown by PLD has not been analyzed in detail up to now. The present study is intending to fill this gap and to show that even small amounts of impurities can significantly influence the electric properties of PZT films grown by PLD, especially the magnitude of the leakage current.

## 2. Materials and Methods

### 2.1. Fabrication of Pure Target

A PZT target, with a nominal composition of Pb(Zr_0.2_Ti_0.2_)O_3_ and 99.9% purity, was acquired from Pi-KEM Ltd, Tamworth, UK. On the other hand, a PZT target of the same composition was manufactured in-house using high purity oxide as precursor materials: PbO 99.999%; ZrO_2_ 99.99%; and TiO_2_ 99.99% from Sigma-Aldrich, Saint-Louis, MO, USA. Ethanol 99.99% purity (Alfa Aesar/Thermo Fisher, Kandel, Germany) was used as the solvent. Homogenization of the powders was performed in a Retsch planetary mill (Retsch Romania, Bucharest, Romania), at 150 rpm for 2 h. Calcination annealing was performed at 850 °C for 2 h, followed by milling for 3 h. The resulting powder was cold pressed using a 40 mm diameter stainless steel mold and a pressure of 19 MPa. The resulted green ceramic disk was sintered in air at 1200 °C for 2 h with a heating rate of 2 °C per minute. The ceramic disk was placed in an alumina crucible enveloped in PbO powder to reduce the Pb losses during sintering. The density of the final ceramic was estimated to about 7.38 g/cm^3^ using the volume and the weight. We underline that neither the commercial target, nor the in-house made target, contained an excess of PbO; thus the only difference was the purity of the starting materials.

### 2.2. Deposition of Thin Films

The PZT thin films from the two targets were grown in a PLD workstation from Surface GmbH, using a KrF excimer laser having 248 nm wavelength, 700 mJ energy, and a maximum 10 Hz repetition rate. The films were deposited on single crystal SrTiO_3_ (STO) substrates with (001) orientation. Prior to deposition, the substrate was cleaned in HF buffer solution and then annealed in air at 1000 °C for 2 h. Smooth terraces of one-unit cell step were obtained in this way [25]. A SrRuO_3_ (SRO) layer of about 20 nm thickness was first deposited by PLD, serving as the bottom electrode, and then the PZT films were grown. The SRO layer was deposited at 700 °C, with a laser fluence of 2 J/cm^2^, a repetition rate of 5 Hz, an oxygen pressure of 0.13 mbar, and a distance of 6 cm between target and substrate. Both PZT films were grown in the same conditions, which were found to be optimum in prior studies [26,27]: 6 cm distance between target and substrate, 575 °C substrate temperature, 2 J/cm^2^ laser fluence, 5 Hz repetition rate, and 0.2 mbar oxygen pressure. Capacitor structures were defined by deposition of a top SRO/Pt (or Au) electrode through a shadow mask of 0.1 × 0.1 mm^2^ area, the Pt (or Au) being deposited on top of the SRO just for a better visualization of the top contacts. The top SRO layer was deposited at room temperature, with the same conditions used for the bottom electrode. The thickness of the top SRO layer was estimated to about 30 nm, with a square resistance of 1000 Ω/cm^2^. The Pt (or Au) layer was deposited by radio-frequency magnetron sputtering.

The denominations of the samples are PZT-CO for the film deposited from the commercial target and PZT-IH for the film deposited from the in-house made target.

### 2.3. Characterization Methods

The structure and morphology of the deposited films were investigated using X-ray diffraction (XRD, Rigaku SmartLab, X-ray source with Cu anode, powered at 40 kV and 40 mA, in parallel beam with Ge (220) monochromator in the incident beam and using a Hypix detector in 0D regime; for the reciprocal space mappings, RSM, a Bruker D8 Advance diffractometer was used, in parallel beam, without a monochromator), transmission electron microscopy (TEM, JEM-ARM 200 F from JEOL, Tokyo, Japan), and piezo-force microscopy (PFM, MFP-3D-SA Asylum Research/Oxford Instruments, Oxford, UK).

Electric measurements at different temperatures were performed by inserting the samples in a Lake Shore cryo-station (Lake Shore Cryotronics, Westerville, OH, USA) with micromanipulated arms and CuBe needles for contacting the SRO electrodes of the ferroelectric capacitor. Dynamic hysteresis loops were recorded using a TF2000 ferristester from Aix ACCT (AixACCT Systems GmbH, Aachen, Germany), capacitance–voltage (C–V) characteristics were measured using an LCR bridge from Hioki (Hioki E.E. Corporation, Nagano, Japan), while current–voltage (I–V) characteristics were recorded using a Keithley 6517B electrometer (Tektronix, Beaverton, OR, USA) with an incorporated dc voltage source.

Laser ablation combined with inductively coupled plasma mass spectrometry (LA–ICP-MS) was used to analyze the elemental composition of all samples included in this study. A New Wave Research laser sampler model NWR-213 (Elemental Scientific Lasers, Bozeman, MT, USA) was used for ablation of the samples. For better reproducibility and to avoid interferences from the bulk, scan analysis through laser ablation of the synthetic laboratory standards and the PZT wafers was used. Analysis of all the standards and samples was conducted using a 40 μm diameter laser spot, 10 J/cm^2^ fluence, and 1 Hz pulse rate, in all cases. The synthetic laboratory standards and the samples were placed on a programmable xyz-translation stage operated via computer control. During laser ablation, the sample surface was monitored using a CCD camera and displayed on a TV screen. After laser ablation, the sample aerosol was transported through Tygon plastic tubing (I.D.: 10 mm; length: 1.0 m) and introduced into the PlasmaQuant MS Elite ICP-MS system from Analytik Jena (Jena, Germany) for detection of the signal intensity of the investigated ions.

Upon connecting the laser ablation system, the synthetic laboratory standards were ablated, and the aerosols were introduced continuously into the ICP-MS system to measure the elements of interest signals. The spot position and focusing position of the laser ablation system were aligned to provide the maximum and most consistent signal with reasonable stability. After a suitable sampling time, the laser ablation system was switched off.

Synthetic laboratory standard materials preparation procedure. In order to calibrate the laser ablation processes and the ICP-MS response, different synthetic laboratory standards were prepared by using only certified reagents. For instance, ultrapure water (type 1: conductivity of 0.055 μS/cm and a resistivity of 18.2 MΩ·cm at 25 °C) that had been deionized using an Evoqua system was used in all preparation procedures. Used as matrix for the elements of interest, polyvinyl alcohol (United States Pharmacopeia Reference Standard) was purchased from Merck and hydrated overnight before deposition. It has to be underlined that this type of matrix comprises only C, H, and O, elements that are not detected by our ICP-MS system as they are mass-excluded elements.

The ICP-MS calibration standard (XXI) with 29 elements (Ag, Al, As, Ba, Be, Bi, Ca, Cd, Co, Cr, Cs, Cu, Fe, Ga, In, K, Li, Mg, Mn, Na, Ni, Pb, Rb, Se, Sr, Tl, U, V, and Zn) with a concentration of 10 mg/L and two standard element solutions (Si, Hf) with a concentration of 1000 mg/L certified reference materials (CRM) were purchased from CPA Chem Switzerland.

Different polyvinyl alcohol solutions containing different elements were prepared by adding certain volumes of CRM solutions. The elements of interest-containing films were deposited onto the glass slides by using a spin coating machine with the following deposition parameters: 1000 rpm for 60 s and 25 deposition steps. After each deposition step, the wafers were dried over a heating plate at 80 °C for 10 min. After subsequent 25 deposition steps, the measured thicknesses of the synthesized films were ~0.8 mm.

## 3. Results

### 3.1. Structural Analysis

**Figure 1:**
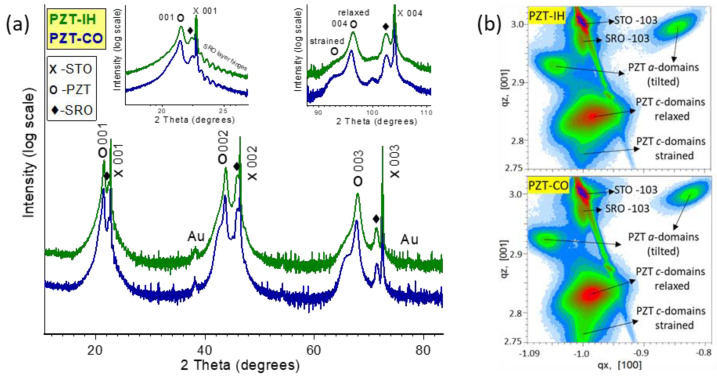
(**a**) The XRD patterns of the samples, showing in the insets details around the 001 and 004 lines of the substrate. (**b**) Reciprocal space mappings in the vicinity of the −103 node of the STO substrate.

The XRD results are presented in Figure 1. One has to mention that XRD analysis was performed on the samples with both electrodes, as the top contacts were deposited immediately after deposition of the PZT layer to avoid contamination of the surface.

For both samples, two types of tetragonal PZT could be observed, with different values for the lattice constant c—perpendicular on the substrate. Most of the films had tetragonal structures with c values close to the bulk one [28,29], while the layers just above the substrate had larger c values. One can consider that the first layers were strained by the substrate, while the rest of the film was partly relaxed, forming a/c domain structures. The out-of-plane parameters were determined from the graphs presented in Figure 1a, and the obtained values were 4.134 Å (relaxed) and 4.236 Å (strained) for PZT deposited from pure target; and 4.145 Å (relaxed) and 4.243 Å (strained) for PZT deposited from the commercial target. The in-plane lattice parameters were determined from the RSM images shown in Figure 1b, obtaining for the relaxed PZT a = 3.98 Å for the film from pure target and a = 3.97 Å for the film from the commercial target. The "strained" domains had the same in-plane lattice constants as the substrate.

The film deposited from the commercial target had a slightly larger value of the c lattice constant. This is already an interesting result, considering that, except for the purity of the targets, all the other parameters were identical (same substrate, same deposition conditions). As polarization is related to the tetragonality factor c/a [30,31,32], and taking into account that the a lattice constants are practically similar, it was found that a larger value for c led to a larger tetragonality, and thus to a larger polarization in the film deposited from the commercial target. One can also presume that the commercial target, containing a larger amount of impurities, had a larger number of free carriers, allowing a better compensation of the depolarization field. This in turn could lead also to a slightly larger polarization, in agreement with the XRD results.

**Figure 2:**
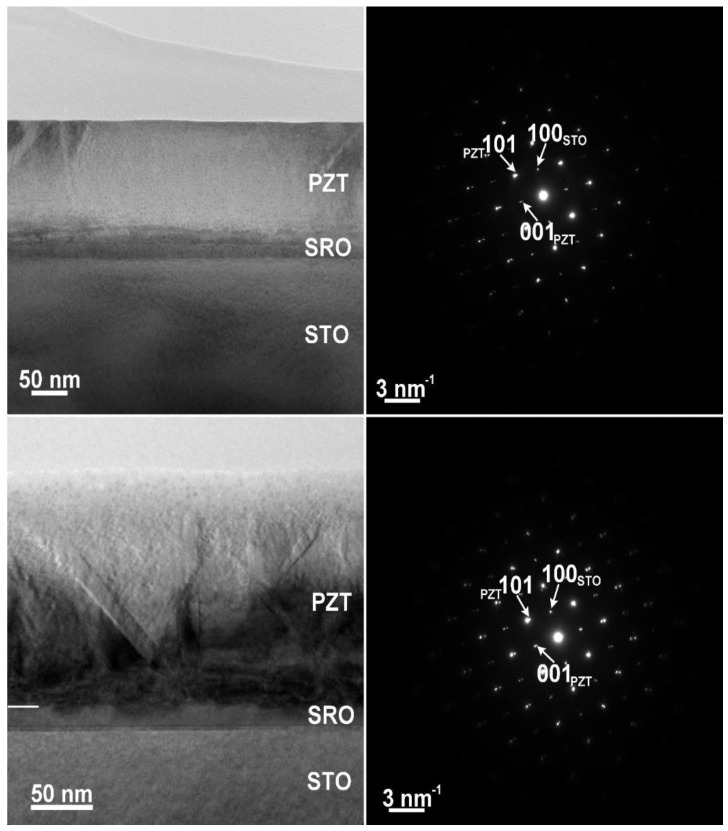
Upper-line: PZT layer grown from the commercial target (PZT-CO); lower-line: PZT layer grown from the in-house manufactured target (PZT-IH).

TEM images are presented in Figure 2, together with selected area electron diffraction (SAED) patterns. grown from the in-house manufactured target (PZT-IH). One can see that the epitaxial quality was very similar, and the crystalline structure was tetragonal in both cases, with some amount of *a*–*c* domains. The presence of *a*–*c* domains was possible due to the thickness of the layers: about 170 nm for PZT-IH, and about 150 nm for PZT-CO. At these thicknesses, the films could relax [33,34] with formation of *a* domains, as revealed by the XRD–RSM measurements.

**Figure 3:**
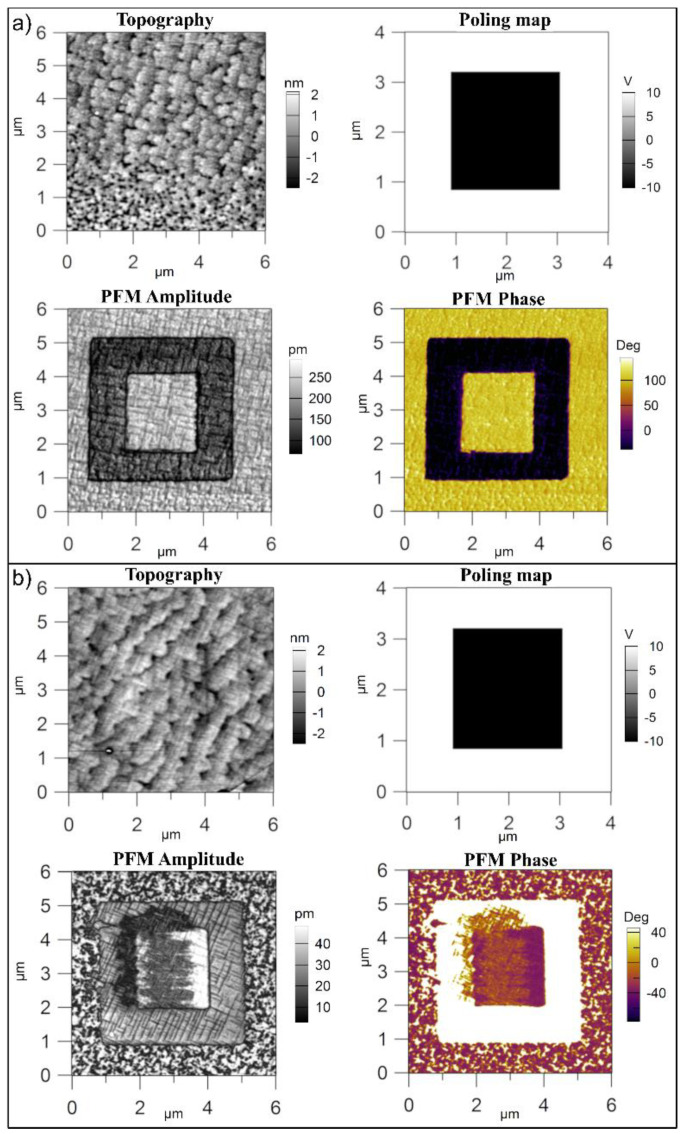
Topography, poling map, PFM amplitude, and phase for (**a**) PZT-CO and (**b**) PZT-IH films.

It appears that the purity of the target did not significantly affect the crystalline structure and the quality of the epitaxial growth. However, PFM investigations, during which a voltage was applied on the sample, started to reveal some differences between the two films, as shown in Figure 3.

Important differences were observed when analyzing the results of the PFM investigations presented in Figure 3. The film deposited from the commercial target had the expected grid of 90 degree domain walls, with a dominant upward direction of the ferroelectric polarization (towards the surface of the film) [27,35]. The film deposited from the pure target showed a more complex domain structure. It appears that 180 degree domains were present in the as-deposited film, as suggested by the phase contrast present on the surface outside the square areas poled with −10 and +10 V. The 90 degree domain grid was recovered after poling, as could be seen in the amplitude image of the inner square and of the outer square-like frame. It appears that the difference in purity had an effect on the domain structure of the as-grown films, probably due to the different amount of free charges available to compensate the depolarization field. In the film grown from the commercial target, there were probably enough free carriers to compensate the depolarization field and to stabilize a dominant upward polarization state, with a small amount of 90 degree domains having in plane polarization. The film deposited from pure target likely had a lower density of free carriers, leading to formation of domains with upward and downward orientations of polarization. The poling process could inject enough carriers to fully compensate the depolarization field and to recover the grid of 90 degree domains.

### 3.2. Electrical Characterization

**Figure 4:**
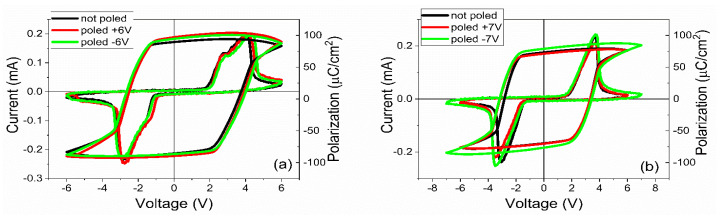
Hysteresis loop obtained at 300 K for the PZT films deposited from the (**a**) commercial target and (**b**) from the pure target. Measurements were performed using a triangular voltage wave of 1 kHz amplitude.

**Figure 5:**
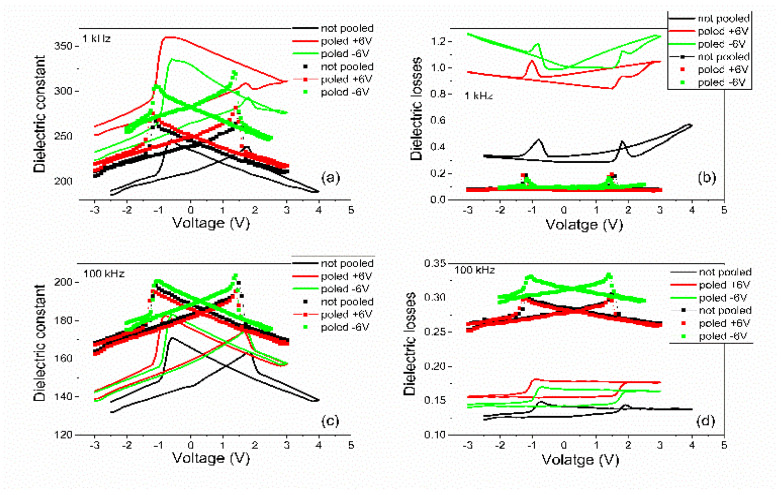
The voltage dependence of the dielectric constant as extracted from the C–V characteristics and the voltage dependence of the dielectric losses, obtained for PZT films grown from commercial target (lines) and from pure target (markers). Measurements performed at 300 K using an ac voltage of 0.1 V amplitude and (**a**,**b**) 1 kHz frequency and for (**c**,**d**) 100 kHz.

**Figure 6:**
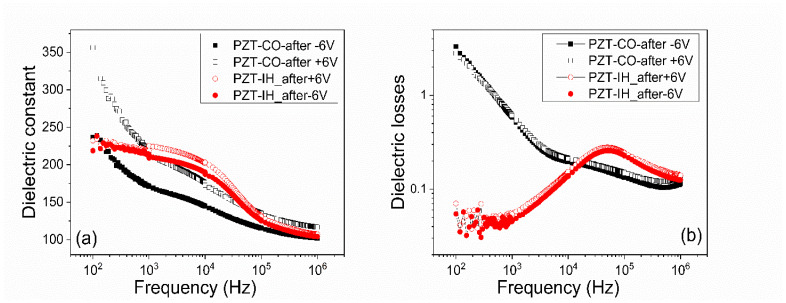
The frequency dependence of (**a**) capacitance and (**b**) dielectric losses for the PZT films deposited from commercial target (PZT-CO) and in-house made, pure target (PZT-IH). The measurements were performed at room temperature using an ac voltage of 0.1 V amplitude. The samples were first poled to fix the polarization state; then the poling voltage was removed, and the measurements started without any dc voltage applied on the sample.

**Figure 7:**
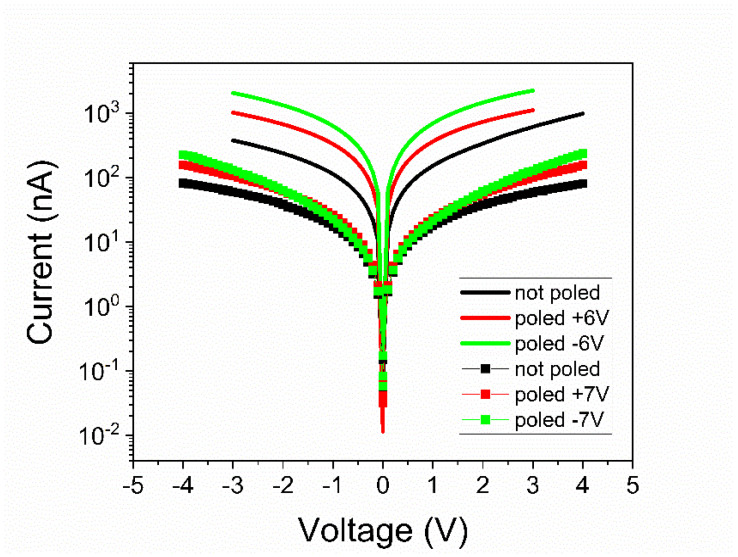
I–V characteristics obtained for PZT films grown from commercial target (lines) and from pure target (markers).

Electrical measurements were further performed. The obtained results are comparatively presented in Figure 4, Figure 5, Figure 6 and Figure 7. All the measurements were performed in three situations: on virgin contact; samples pre-poled with a negative voltage applied on the top contact at room temperature, leading to an initial upward polarization state; and samples pre-poled with a positive voltage applied on the top contact at room temperature, leading to an initial downward polarization state.

Analyzing Figure 4, Figure 5, Figure 6 and Figure 7 one can observe the following:

- The polarization values were around 75–80 µC/cm^2^ for PZT deposited from pure target (PZT-IH) and about 85–90 µC/cm^2^ for the film deposited from commercial target (PZT-CO). This is in agreement with the findings of the XRD analysis, showing a larger value for the out-of-plane lattice constant in the case of PZT-CO compared to PZT-IH.
- The dielectric constant had different values depending on the polarization state. However, the dispersion of the values seemed to be larger for the PZT film deposited from the commercial target. One can notice that, in the case of PZT-CO, the complete butterfly shape at 1 kHz was obtained only for the contact that was not poled initially. For the other two cases, with upward or downward poling, there were large differences between the magnitudes of the dielectric constant when the voltage was swept up or down. Complete butterfly shapes were obtained at 100 kHz for both samples. An internal electric field was present, as suggested by the fact the C–V characteristics were shifted towards positive voltages for both PZT-IH and PZT-CO. This field was oriented towards the top electrode in all cases, in agreement with the polarization hysteresis loops presented in Figure 4 and showing also a shift towards the positive voltage. This fact suggests that the origin of the internal field might be the same and related to the presence of the strained layer just above the substrate, at the beginning of the epitaxial growth. In fact, the presence of strained and more relaxed layers in both PZT films suggests the presence of a strain gradient, which can lead to the presence of a flexoelectric internal field [36,37,38,39].
- The dielectric losses at 1 kHz were much larger for PZT-CO compared to PZT-IH, suggesting a larger dc conduction (larger leakage current). As in the case of the dielectric constant, the magnitude of the losses depended significantly on the polarization orientation and state (poled or not poled). At 100 kHz, the losses were larger for PZT-IH. However, it is beyond the scope of this study to investigate in detail the dependence of the dielectric properties on the polarization state and orientation, as well as on frequency. One can just assume that the impurity atoms were introducing levels in the gap that could be filled or not depending on polarization orientation (especially near the electrode interfaces, where the band bending occurs), and that could influence the frequency response depending on their presence in the sample.
- The frequency dependence of the dielectric constant (Figure 6a) showed a continuous decrease of value for the PZT film deposited from the commercial target, while the film deposited from the pure target (PZT-IH) showed a relatively constant dielectric constant up to about 10 kHz, and then a decrease towards a value comparable to that of the PZT-CO sample. The results were in agreement with those obtained from C–V measurements. The losses for PZT-CO were very large at low frequencies, also in agreement with the results presented in Figure 5b, suggesting high dc leakage current. The losses for PZT-IH showed a relaxation peak at around 50–60 kHz. The losses for PZT-IH became larger than for PZT-CO at frequencies above 10 kHz, in agreement with the results presented in Figure 5c. The differences can be attributed to different content of impurities, triggering different distribution of energy levels in the gap. However, it is beyond the scope of the present study to identify the nature of the impurities and the position of the associated energy levels in the gap.
- The magnitude of the leakage current decreased significantly for the film grown from the pure target, statistically with more than one order of magnitude. Statistics performed on several electrodes, randomly selected, revealed that the leakage current, could have, sometimes, a significant dependence on the polarization state, more precisely on the polarization orientation, as presented in Figure 7. However, in most cases, the difference was small or absent. Statistically it was also observed that the current magnitude was larger after poling the samples compared to currents measured on not poled samples (as grown).

Considering the above results, one may conclude that the target purity has an important effect on the electric and dielectric properties of epitaxial PZT thin films. As previously mentioned, the commercial target had 99.9% purity, while the precursor oxides for the in-house made target were of at least 99.99% purity. Assuming some accidental impurification during the milling process, still the purity of the in-house made target was better, possibly with up to one order of magnitude, than that of the commercial target. The result is that the impurities from the commercial target may have played the role of donors or acceptors, introducing a larger density of free carriers compared to the in-house made target. This can explain the larger magnitude of the leakage current from the PZT film grown from the commercial target. One can estimate the maximum concentration of free carriers that can be introduced by the impurities from the commercial target acting as donors or acceptors, knowing that the lattice constant is about 4 × 10^−10^ m and assuming an impurity atom at each 10,000 unit cells (the equivalent of a difference of 0.01% in purity), which results in a density of about 1.5 × 10^24^ m^−3^.

C–V and I–V measurements were performed at different temperatures, and the data were used to extract information about the effective density of carriers N_eff_ and potential barriers at zero applied voltage *Φ_B_*^0^ at the electrodes for different polarization states (not poled, fully upward or fully downward). The procedures are those previously presented in the literature [40,41], and the intermediate steps and graphs are presented in the Supplementary Materials.

The results are synthesized in Table 1 for N_eff_ (at 100 K and 300 K), and in Table 2 for *Φ_B_*^0^.

One can consider that the error for estimation of N_eff_ was quite large, of about 30–50% thus, coming mostly from the derivative of the 1/C^2^ plot (see also [26]). Within these errors, one can conclude that the density of free carriers was about the same in the PZT films deposited from pure and commercial targets. This is rather odd, taking into account the large difference in the magnitude of the leakage current. However, the leakage current is measured for a given state of polarization and is given by the free carriers that still can move under the applied electric field. N_eff_ is extracted from a C–V characteristic involving polarization switching; thus, these are the charges from the film (free carriers, ionized donors or acceptors, trapped charges) that are contributing to the compensation of polarization charges, together with the free carriers from the electrodes. As the polarization values are close in magnitude, one can expect similar values for N_eff_ in the case of the two samples. If one compares the values in Table 1 with the estimation of free carrier concentration in the commercial target, one can see that this concentration is not enough to compensate the polarization charge resulted from the hysteresis measurements, and thus, the main contribution is coming from the self-doping [27] and electrodes.

Regarding the potential barriers *Φ_B_*^0^ presented in Table 2, one can observe the followings:

- The potential barriers for the PZT film deposited from commercial target were rather similar, no matter the initial polarization state. The difference for positive and negative branches of the I–V characteristics was larger only for the as-deposited sample, and this was because there might have been defects that could migrate under the applied electric field, affecting the interface properties. Once the polarization state was clearly defined and there were no longer migrating defects, the potential barrier stabilized at around 0.11–0.13 eV for both voltage polarities. It has to be mentioned that the error in estimating the potential barrier was of about 0.015 eV; thus, the values presented in Table 2 are within these errors.
- The results were similar for the film deposited from the pure target; the as deposited films had larger barriers, but these dropped to about 0.140–0.155 eV once the ferroelectric polarization had a clear orientation in the film. Nevertheless, the potential barriers were constantly higher compared to those obtained for the film deposited from the commercial target. The difference was between 0.020 eV and 0.070 eV depending on the polarization state. On average, the potential barriers were with about 0.04–0.05 eV higher for the film deposited from the pure target. Assuming that the Schottky emission was the dominant conduction mechanism around room temperature, the results are that this difference in the height of the potential barriers at the electrode interfaces could produce a difference of at least one order of magnitude between the leakage currents, with a lower value in the film deposited from the pure target. This is in good agreement with the experimental results presented in Figure 7.
- One can note that the heights of potential barriers were comparable to previous reports in the literature [26]. One can argue that these values were considerably lower than those extracted from X-ray photoelectron spectroscopy (XPS) measurements, which were around 1 eV [43]. However, if one considers the polarization contribution to the reduction of the potential barrier [42], the estimated values increased to about 0.6 eV (a value obtained when using a static dielectric constant of about 200, an average polarization value of about 80 µC/cm^2^, and an optical dielectric constant of about 6.5 [42] (see also Supplemental Information), a value that started to be comparable to the values extracted from XPS. The issue for discussion is which value should be used for the static dielectric constant, the one extracted from capacitance measurements (see Figure 5) or the intrinsic value, estimated to be between 30 and 40 [44]. If a value of 40 is used for the static dielectric constant, then the height of the potential barrier becomes about 0.8–0.9 eV, much closer to the values estimated from XPS data. In any case, the topic of the dielectric constant in ferroelectrics is beyond the scope of the present article.

### 3.3. Chemical Analysis

A great effort was made to obtain information about the chemical composition of the targets, assuming that PLD is a method that translates the target stoichiometry into the grown film. Therefore, it was expected that the impurities from the target were to be found in the film also. The starting point was the certificate of analysis from the target provider, in the case of the commercial one, and the certificates of analysis for the starting oxides used to prepare the in-house “pure” target. The trace elements, as evidenced by inductively coupled plasma mass spectroscopy (ICP-MS) analysis performed by the suppliers, are presented in Table 3. In the same table are also presented the possible valences and the ionic radii.

Several remarks can be drawn by analyzing Table 3:

- If one adds the estimated quantities for all trace elements, one obtains about the same total, around 130–140 ppm weight, although the declared purity of the commercial target is 99.9% by weight and the purity of the starting metal oxides for the pure target is at least 99.99% by weight. This result suggests that the commercial target may contain other impurities that could not be detected by the method used to assess the target purity.
- There are isovalent impurities, such as Ba, Ca, Cd, Sr, Hf, Si, and Sn, which can replace Pb or Ti/Zr without introducing free carriers in the sample.
- Aside from Pb and Ti that are present in the matrix, some traces of Pb and Ti were detected in the targets, but it is not clear if these traces are related to some unreacted PbO and TiO_2_ quantities.
- The other elements found in the targets are of different valance compared to Pb and Ti/Zr and can replace these elements in the PZT matrix if they fulfill the Goldschmidt tolerance factor [46], or the newly defined tolerance factor in [47]. In any case, these heterovalent impurities can act as donors or acceptors in PZT, thus affecting the density of the free carriers in the sample. Eliminating the isovalent elements, then the amount of heterovalent elements, those that can introduce free carriers in the PZT layer, is about 95 ppm in the commercial target and about 19 ppm in the pure target manufactured in house from as purse as possible precursor oxides. The difference can justify the larger leakage current in the film deposited from the commercial target, as well the larger polarization value due to a better compensation of the depolarization field. They can affect also the lattice constant, due to different ionic radii, and can introduce a multitude of energy levels in the PZT’s gap, affecting the position of the Fermi level and the height of the potential barriers at the electrode interfaces.

A second check was to perform in-house ICP-MS measurements using the equipment and standards described in Section 2, namely C-characterization methods. The results are synthesized in Table 4.

Excluding the isovalent elements, one can easily verify that the concentration of the heterovalent elements that can introduce donor or acceptor levels in PZT is of about two times larger in the commercial target compared to the pure one, about 5.2 × 10^25^ m^−3^ compared to about 2.22 × 10^25^ m^−3^. The difference in concentration is larger than the one estimated from the 0.09% difference in the purities of the two targets and is comparable to the values estimated in Table 1 from C–V measurements. In any case, one has to consider that the heterovalent impurities detected in the targets can act as donor or acceptor levels in the gap depending on which atom they substitute, Pb or Zr/Ti, respectively. Depending on the valence state, the ionic radii are smaller or larger, being closer to that of Pb or that of Ti/Zr. Therefore, it is difficult to estimate the number of acceptors or donors coming from the unwanted impurities that are present in the targets, and to extract a net concentration of free carriers, not to mention that oxygen vacancies and, possibly, lead vacancies can be present. Other defects, as the recently suggested anti-site defect [48], can be also present, adding more variables to the real density of free carriers in the PZT. What is clear from the experiments is that in the commercial target, more impurities can act as donors and acceptors compared to the pure targets, which can explain all the differences observed in the electrical measurements.

## 4. Conclusions

Epitaxial Pb(Zr_0.2_Ti_0.2_)O_3_ films were deposited by PLD from targets of different purities, namely 99.9% (commercial target) and 99.99% (in-house made target), respectively. Their structural and electrical properties were investigated, and several significant differences were found:

- The *c* lattice constant is with about 0.15% larger in the film deposited from the commercial target compared to the film deposited from the in-house made target, leading to a larger tetragonality and larger value of polarization, namely 85–90 µC/cm^2^ in PZT-CO compared to 75–80 µC/cm^2^ in PZT-IH.
- The PZT-CO has an upward orientation of polarization, with the characteristic grid of 90 degree domain walls, while the PZT-IH shows a structure of mixed domains, 180 degree domains being also present. These differences in domain structure reflect in different values of the dielectric constant, which is larger in PZT-IH.
- The leakage current is about one order of magnitude lower in PZT-IH, while the potential barriers at electrode interfaces are about 40–50 meV higher compared to PZT-CO.
- Composition analysis revealed that the amount of heterovalent impurities is about two times larger in the commercial target, with impacts on the density of free carriers, on the position of the Fermi level, and on the magnitude of the leakage current.

In conclusion, the target purity can significantly affect the macroscopic electrical properties of PZT thin films. This aspect has to be taken into consideration when doping studies are performed because it will be hard to distinguish between the effect of the unwanted impurities coming from the target and the intentionally introduced impurities to act as donors or acceptors.

Further studies are needed to accurately quantify the metal trace impurities that can act as donors or acceptors in ferroelectric PZT thin films. This can be done using well-calibrated references for ICP-MS measurements. A further step is to reduce as much as possible the amount of unwanted impurities, eventually trying to further purify the commercial raw materials, although this can be a difficult and time-consuming task. However, this effort can be rewarding, allowing accurate control of doping for developing true ferroelectric p–n homojunctions.

## Figures and Tables

**Table 1:** The effective density of charges N_eff_ extracted from the C–V characteristics recorded at different temperatures (similar to a metal-semiconductor Schottky contact, can be considered as the density of charge in the depleted region [41,42]).

Sample		N_eff_ (m^−3^)			
		100 K		300 K	
		Positive Voltage	Negative Voltage	Positive Voltage	Negative Voltage
PZT-IH	AD	9.093 × 10^24^	8.83 × 10^24^	9.9 × 10^24^	9.61 × 10^24^
	P+	8.16 × 10^24^	9.92 × 10^24^	1.013 × 10^25^	9.69 × 10^24^
	P−	8.89 × 10^24^	8.55 × 10^24^	1.0269 × 10^25^	9.84 × 10^24^
PZT-CO	AD	7.05 × 10^24^	8 × 10^24^	6 × 10^24^	7.17 × 10^24^
	P+	7.35 × 10^24^	6.54 × 10^24^	9.58 × 10^24^	8.15 × 10^24^
	P−	9.58 × 10^24^	8.8 × 10^24^	8.17 × 10^24^	6.52 × 10^24^

**Table 2:** The potential barrier at zero volt *Φ_B_*^0^ extracted from the I–V characteristic recorded at different temperatures following the procedure from [41,42].

Sample		$\Phi_{B}^{0V}\;\left(eV\right)$	
		Positive Voltage	Negative Voltage
PZT-IH	AD	0.285	0.273
	P+	0.136	0.153
	P−	0.153	0.149
PZT-CO	AD	0.113	0.209
	P+	0.118	0.114
	P−	0.127	0.121

**Table 3:** Trace elements detected in the commercial target and in the metal oxides used as raw materials for the pure target (data from suppliers). For some elements, the quantity in ppm could be estimated. ND is for not detected or below the sensitivity limit of the equipment. The possible valance states are also given, together with the corresponding ionic radii [45].

Elements	Commercial Target (ppm Weight)	Pure Target (ppm Weight)	Valence State	Ionic Radii (pM)
Ag	<1	ND	1+/2+/3+	129/108/89
Al	ND	10	3+	67.5
As	<4	11.5	3+/5+	72/68
B	ND	0.4	3+	41
Ba	2	1	4+	149
Bi	<15	ND	3+/5+	117/90
Ca	ND	17.4	2+	114
Cd	<3	17	2+	109
Cl	<8	ND	1−	167
Co	<1	ND	2+/3+	79/68.5
Cr	<1	ND	2+/3+/4+/5+/6+	87/75.5/69/63/58
Cu	<2	ND	1+/2+	91/87
Fe	<4	5.2	2+/3+	92/78.5
Hf	<10	ND	4+	85
K	1	50.7	1+	152
Mg	2	1.1	2+	86
Mn	<1	0.2	2+/3+	97/78.5
N	<27	ND	3−	132
Na	<10	8	1+	116
Ni	<4	ND	2+/3+/4+	83/70/62
Pb	1	6.9	2+/4+	133/91.5
S	<2	ND	2−	170
Sb	<4	ND	3+/5+	90/74
Se	<1	ND	4+/6+	64/56
Si	<10	6	4+	54
Sn	<4	ND	4+	83
Sr	2	ND	2+	132
Te	<3	ND	4+/6+	111/70
Ti	2	ND	3+/4+	81/74.5
V	ND	2	2+/3+/4+/5+	93/78/72/68
Zn	<3	2.3	2+	88
Zr	ND	ND	4+	86

**Table 4:** Concentration of elements detected in the commercial and pure targets using ICP-MS analysis.

Commercial Target		
Symbol	Element	Concentration (at/cm^3^)
Ag	Silver	1.38 × 10^19^
Al	Aluminum	2.04 × 10^19^
Bi	Bismuth	3.09 × 10^17^
Cr	Chromium	2.29 × 10^18^
Mg	Magnesium	4.25 × 10^18^
Mn	Manganese	9.96 × 10^18^
Ni	Nickel	1.29 × 10^18^
Si	Silicon	9.47 × 10^18^
Sr	Strontium	3.5 × 10^18^
Zn	Zinc	1.33 × 10^17^
**Pure Target**		
**Symbol**	**Element**	**Concentration (at/cm^3^)**
Al	Aluminum	1.12 × 10^19^
Bi	Bismuth	4.56 × 10^17^
Cr	Chromium	2.83 × 10^17^
Mn	Manganese	9.15 × 10^18^
Ni	Nickel	1.59 × 10^18^
Si	Silicon	7.64 × 10^18^
Sr	Strontium	3.16 × 10^18^
Zn	Zinc	9.14 × 10^17^

## Data Availability

The data that support the findings of this study are available from the corresponding author upon reasonable request.

## Supplementary Materials

The following are available online at https://www.mdpi.com/article/10.3390/nano11051177/s1. In the Supplementary Material are shown examples of how the values for N_eff_ and *Φ_B_^0^* were estimated. The data that support the findings of this study are available within the article, its Supplementary Material, or from the corresponding author upon reasonable request.
