# Supplementary material for: Accidental Impurities in Epitaxial Pb(Zr0.2Ti0.8)O3 Thin Films Grown by Pulsed Laser Deposition and Their Impact on the Macroscopic Electric Properties
Source: Nanomaterials (Basel). 2021 Apr 29;11(5):1177. doi: 10.3390/nano11051177 (PMC8146871; doi:10.3390/nano11051177)
Supplement: Supplementary file 1 [file nanomaterials-11-01177-s001.zip › nanomaterials-1194211-supplementary.pdf]

Supplementary Material

Accidental Impurities in Epitaxial $Pb(Zr_{0.2}Ti_{0.8})O_3$ Thin Films Grown by Pulsed Laser Deposition and Their Impact on the Macroscopic Electric Properties

Georgia Andra Boni, Cristina Florentina Chirila, Viorica Stancu, Luminita Amarande, Iuliana Pasuk, Lucian Trupina, Cosmin Marian Istrate, Cristian Radu, Andrei Tomulescu, Stefan Neatu, Ioana Pintilie, Lucian Pintilie *

National Institute of Materials Physics, Atomistilor 405A, 077125 Magurele, Romania; andra.boni@infim.ro (G.A.B.); dragoi@infim.ro (C.F.C.); stancu@infim.ro (V.S.); amarande@infim.ro (L.A.); iuliana.pasuk@infim.ro (I.P.); Lucian.Trupina@infim.ro (L.T.); cosmin.istrate@infim.ro (C.M.I.); cristian.radu@infim.ro (C.R.); andrei.tomulescu@infim.ro (A.T.); stefan.neatu@infim.ro (S.N.); ioana@infim.ro (I.P.)

* Correspondence: pintilie@infim.ro

1. Determination of N_{eff}

The following deduction was performed considering that ferroelectric PZT is a wide gap semiconductor, and the ferroelectric-metal contacts are Schottky-type contacts. Thus, a metal-ferroelectric-metal (MFM) structure could be considered as two Schottky diodes connected back-to-back. One of the diodes will be reverse biased, whatever the polarity of the applied voltage, and in this way the capacitance of the MFM structure can be defined (for voltage ranges where the polarization is totally reversed) [1]:

$$\frac{C}{A} = \frac{\varepsilon_0 \varepsilon_{st}}{w} = \sqrt{\frac{q\varepsilon_0\varepsilon_{st}N_{eff}}{2(V+V'_{bi})}} \quad (1)$$

where A is the geometrical area defined by the top metallic contact, $\boldsymbol{\varepsilon_0}$ is the permittivity of the free space, $\boldsymbol{\varepsilon_{st}}$ is static dielectric constant, $\boldsymbol{q}$ is the elementary charge, $\boldsymbol{N_{eff}}$ is the effective charge density, $\boldsymbol{V}$ is the applied voltage, $\boldsymbol{w}$ is the depletion layer width, and $\boldsymbol{V'_{bi}}$ is the apparent builtin potential.

The $\boldsymbol{N_{eff}}$ values are determined by the derivative of the representation of $\frac{1}{C^2} = f(V)$. Thus,

$$N_{eff} = \frac{2}{q\varepsilon_0\varepsilon_{st}[d(1/C^2)/dV]}. \quad (2)$$

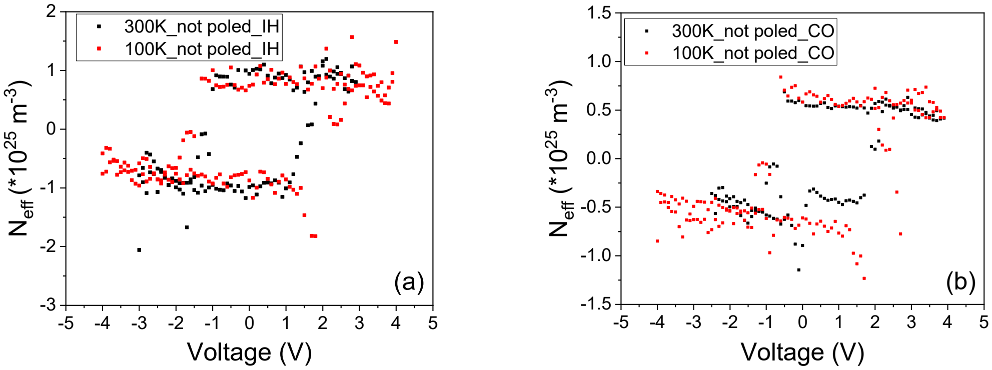

Figure S1. Representation of determination of N_{eff} for 100 K and 300 K in the case of the (**a**) PZT-IH sample and the (**b**) PZT-CO sample.

2. Determination of the potential barrier

The dominant conduction mechanism is considered to be thermionic emission with the mean free path of the charge carriers in PZT being much smaller than the film thickness; thus, the current density, J, is described by the Schottky–Simmons equation [2] (the injection is limited by the potential barriers at the electrode interfaces, and the drift–diffusion in the film is limited by the carrier's mobility) [1,3,4]:

$$J = 2q\left(\frac{2\pi m_{eff}kT}{h^2}\right)^{3/2}\mu E \exp\left(-\frac{q}{kT}\left(\phi_B^0 - \sqrt{\frac{qE}{4\pi\varepsilon_0\varepsilon_{op}}}\right)\right) \quad (3)$$

where h is Planck's constant, m_{eff} is the effective mass, $\boldsymbol{\mu}$ is the mobility, E is the applied electrical field, k is Boltzmann's constant, $\boldsymbol{\varepsilon_{op}}$ is the optical dielectric constant, $\boldsymbol{\phi_B^0}$ is the interfacial potential barrier height at zero volts, and T is the temperature.

From the slope of the Arrhenius plot, $\mathbf{ln}\left(\boldsymbol{J/T^{\frac{3}{2}}}\right) = \boldsymbol{f}(\mathbf{1000}/\boldsymbol{T})$ as it is represented in Figure S2b. For PZT-CO poled with −6V at room temperature, the apparent potential barrier is obtained for different applied voltages, $\boldsymbol{\phi_B} = \boldsymbol{\phi_B^0} - \sqrt{\frac{\boldsymbol{qE}}{\boldsymbol{4\pi\varepsilon_0\varepsilon_{op}}}}$. Then, $\boldsymbol{\phi_B^0}$ is evaluated from the intercept of the linear dependence of the $\boldsymbol{\phi_B} = \boldsymbol{f}(\sqrt{\boldsymbol{V}})$, as is presented in Figure S2c.

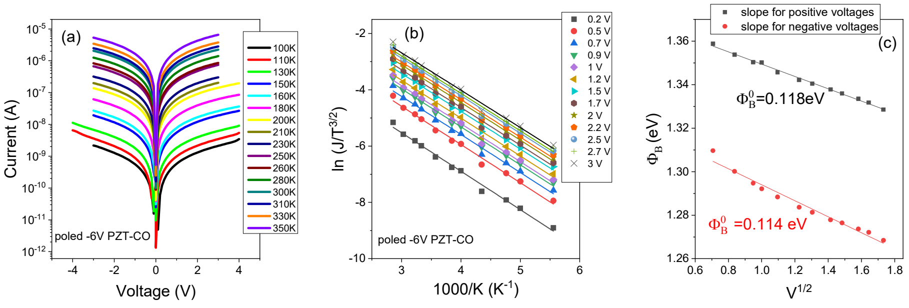

Figure S2. (**a**) Current–voltage measurements for different temperatures; (**b**) the Arrhenius plot for positive voltages; (**c**) the linear representation of apparent potential barrier as function of $\sqrt{\boldsymbol{V}}$ for the cases of PZT-CO poled with −6V at room temperature.

References

1. Pintilie, L.; Alexe, M. Metal-Ferroelectric-Metal Heterostructures with Schottky Contacts. I. Influence of the Ferroelectric Properties. *Journal of Applied Physics* **2005**, *98*, 124103, doi:10.1063/1.2148622.
2. Simmons, J.G. Effect of Deep Traps on the Barrier Heights of Metal-Insulator-Metal Tunnel Junctions. *Phys. Rev. Lett.* **1969**, *23*, 297–300, doi:10.1103/PhysRevLett.23.297.
3. Pintilie, L.; Vrejoiu, I.; Hesse, D.; LeRhun, G.; Alexe, M. Ferroelectric Polarization-Leakage Current Relation in High Quality Epitaxial Pb(Zr,Ti)O3 Films. *Phys. Rev. B* **2007**, *75*, 104103, doi:10.1103/PhysRevB.75.104103.
4. Chirila, C.; Boni, A.G.; Pasuk, I.; Negrea, R.; Trupina, L.; Rhun, G.L.; Yin, S.; Vilquin, B.; Pintilie, I.; Pintilie, L. Comparison between the Ferroelectric/Electric Properties of the PbZr0.52Ti0.48O3 Films Grown on Si (100) and on STO (100) Substrates. *J Mater Sci* **2015**, *50*, 3883–3894, doi:10.1007/s10853-015-8907-2.
